# Crystal structure of *Mycobacterium tuberculosis* FadB2 implicated in mycobacterial β-oxidation

**DOI:** 10.1107/S2059798318017242

**Published:** 2019-01-08

**Authors:** Jonathan A. G. Cox, Rebecca C. Taylor, Alistair K. Brown, Samuel Attoe, Gurdyal S. Besra, Klaus Fütterer

**Affiliations:** a School of Biosciences, University of Birmingham, Edgbaston, Birmingham B15 2TT, England

**Keywords:** *Mycobacterium tuberculosis*, l-3-hydroxyacyl-CoA dehydrogenase, mycobacterial β-oxidation, X-ray crystallography

## Abstract

The intracellular pathogen *Mycobacterium tuberculosis* is the causative agent of tuberculosis, which is a leading cause of mortality worldwide. The survival of *M. tuberculosis* in host macrophages through long-lasting periods of persistence depends, in part, on breaking down host cell lipids as a carbon source. The critical role of fatty-acid catabolism in this organism is underscored by the extensive redundancy of the genes implicated in β-oxidation (∼100 genes). In a previous study, the enzymology of the *M. tuberculosis* l-3-hydroxyacyl-CoA dehydrogenase FadB2 was characterized. Here, the crystal structure of this enzyme in a ligand-free form is reported at 2.1 Å resolution. FadB2 crystallized as a dimer with three unique dimer copies per asymmetric unit. The structure of the monomer reveals a dual Rossmann-fold motif in the N-terminal domain, while the helical C-terminal domain mediates dimer formation. Comparison with the CoA- and NAD^+^-bound human orthologue mitochondrial hydroxyacyl-CoA dehydrogenase shows extensive conservation of the residues that mediate substrate and cofactor binding. Superposition with the multi-catalytic homologue *M. tuberculosis* FadB, which forms a trifunctional complex with the thiolase FadA, indicates that FadB has developed structural features that prevent its self-association as a dimer. Conversely, FadB2 is unable to substitute for FadB in the tetrameric FadA–FadB complex as it lacks the N-terminal hydratase domain of FadB. Instead, FadB2 may functionally (or physically) associate with the enoyl-CoA hydratase EchA8 and the thiolases FadA2, FadA3, FadA4 or FadA6 as suggested by interrogation of the STRING protein-network database.

## Abstract 2

The catabolism of fatty acids in mycobacteria involves an extensively redundant set of enzymes. Here, the structure of the l-3-hydroxyacyl-CoA dehydrogenase FadB2 is described and structural cues as to how it may work with other components of β-oxidation are explored.

## 1. Introduction   

The bacterial pathogen *Mycobacterium tuberculosis* (*Mtb*) is the ninth leading cause of mortality worldwide and remains the foremost cause of death caused by a single infectious agent (World Health Organization, 2018 ▸). Over millennia of co-evolution with its human host (Gagneux, 2012 ▸), the tubercle bacillus has fine-tuned strategies for survival inside host macrophages, its most frequent niche. Infected macrophages trigger the immune system to form a granuloma, a cluster of infected macrophages surrounded by foamy macrophages, lymphocytes and a fibrous cuff (Russell, 2007 ▸). Inside the granuloma, *Mtb* evades immune clearance and enters a long-lasting latency or persistence state. An obvious drawback of this survival strategy is that the organism must cope with an environment in which nutrient supply is scarce and, as a key adaptation, *Mtb* utilizes host cell lipids as a carbon source (Pandey & Sassetti, 2008 ▸; Lee *et al.*, 2013 ▸; Bonds & Sampson, 2018 ▸). Recent evidence shows that *Mtb* degrades host cell cholesterol during persistence (Pandey & Sassetti, 2008 ▸; Wipperman *et al.*, 2014 ▸) and that the inhibition of cholesterol degradation could provide a potential route to control the growth of *Mtb* in macrophages (VanderVen *et al.*, 2015 ▸). The products of cholesterol catabolism include acyl-CoA fatty acids, which are broken down by β-oxidation to acetyl-CoA, feeding into the tricarboxylic acid cycle and gluconeogenesis via the glyoxylate shunt (Pandey & Sassetti, 2008 ▸).

**Figure 1:**
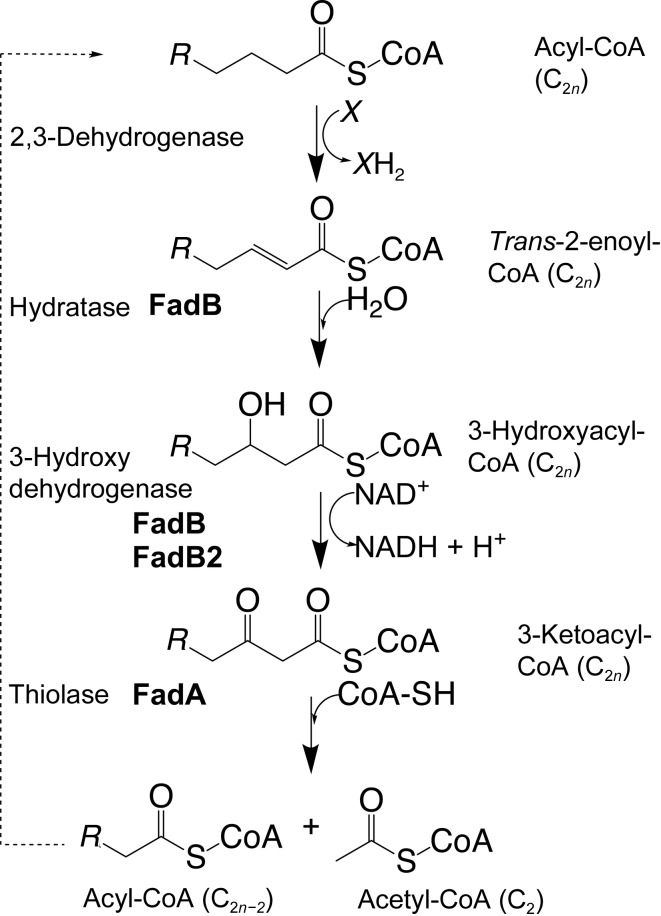
Schematic representation of the reaction cycle for the β-oxidation of CoA-linked fatty acids. In each cycle the CoA-linked acyl chain is shortened from C_2*n*_ to C_2*n*−2_.

The β-oxidation pathway was first described for *Escherichia coli* (Black & DiRusso, 1994 ▸), which has a single set of genes in the fatty-acid degradation (*fad*) operon (Muñoz-Elías & McKinney, 2006 ▸). The pathway requires five distinct enzymatic activities: attachment of (saturated) fatty acids to coenzyme A (CoA) by FadD (acyl-CoA synthetase), followed by catabolism of the CoA-linked acyl chains through an iterative cycle of four reactions (Fig. 1 ▸), with each iteration removing two C atoms from the acyl chain. The cycle begins by introducing a double bond between C2 and C3 (α and β positions), which is followed by hydroxylation (step 2) and hydroxy dehydro­genation at C3 (step 3); the cycle is completed by the thiolytic cleavage of 3-ketoacyl-CoA to give acyl-CoA and acetyl-CoA (step 4).

In contrast to the single set of *fad* genes in *E. coli* (Muñoz-Elías & McKinney, 2006 ▸), the genome of *Mtb* includes about 100 genes linked to β-oxidation (Cole *et al.*, 1998 ▸). This repertoire includes five genes with the *fadB* designation: *fadB* (Rv0860) and *fadB2*–*fadB5*. *Mtb* FadB is a multi-catalytic enzyme that catalyses both the hydration and the 3-hydroxy dehydrogenation steps in the β-oxidation cycle (Venkatesan & Wierenga, 2013 ▸). In contrast, FadB2 (Rv0468) and FadB3 (Rv1715) are smaller, monofunctional enzymes that encompass only a hydroxyacyl-CoA dehydrogenase (HAD) domain. The amino-acid sequences of FadB4 (Rv3141) and FadB5 (Rv1912c) are similar to each other, but show a distinctly lower similarity to the other three enzymes (Taylor *et al.*, 2010 ▸). In a previous study, we characterized the enzymatic properties of *Mtb* FadB2, demonstrating that it harbours 3-hydroxy dehydrogenase activity as well as catalysing the reverse reaction from acetoacetyl-CoA to 3-hydroxyacyl-CoA (Taylor *et al.*, 2010 ▸). Genetic knockout of *fadB2* in *M. smegmatis* had no discernible phenotype, in line with the presence of several *fadB* homologues in the mycobacterial genome. The expression of FadB2 has been observed to be moderately up-regulated under acidic conditions (Fisher *et al.*, 2002 ▸), whereas in an *in vitro* activity assay low pH favoured the reverse reaction, converting acetoacetyl-CoA to 3-hydroxyacyl-CoA, while maximal activity for the forward reaction occurred at pH 10 (Taylor *et al.*, 2010 ▸).

Here, we report the crystal structure of *Mtb* FadB2 at 2.1 Å resolution in the ligand-free form and we use the structure to undertake a structural comparison of mycobacterial FadB homologues.

## 2. Materials and methods   

### 2.1. Cloning, expression and purification of *Mtb* FadB2   

The protocol for obtaining His-tagged recombinant FadB2 has been described previously (Taylor *et al.*, 2010 ▸). Briefly, the gene sequence of *fadB2* (Rv0468) was amplified from *Mtb* H37Rv chromosomal DNA by PCR and the amplified DNA fragment was ligated into the NdeI and HindIII restriction sites of the pET-28b plasmid vector (Novagen). The resulting expression plasmid was heat-shock transformed into *E. coli* C43 (DE3) cells, which were grown on LB agar plates, selecting for transformants with 25 µg ml^−1^ kanamycin. Bacterial colonies were propagated in overnight liquid cultures (5 ml LB, 37°C, 180 rev min^−1^) and used to inoculate bulk cultures (1 l Terrific Broth, 25 µg ml^−1^ kanamycin). Bulk cultures were grown to an OD_600_ of 0.6 (37°C, 180 rev min^−1^) and protein expression was induced by adding IPTG (1 m*M* final concentration). The induced cultures were incubated (16°C, 180 rev min^−1^) and the cells were harvested after 12–14 h by centrifugation (4000 rev min^−1^, 4°C). Cleared cell extracts were prepared in lysis buffer (0.05 *M* sodium phosphate pH 8.0, 0.3 *M* NaCl, 10 m*M* imidazole) supplemented with EDTA-free protease-inhibitor cocktail (Roche) and were purified using Ni–NTA affinity chromatography. The protein was eluted using a step gradient increasing the imidazole concentration in the lysis buffer to 50 m*M*, 130 m*M*, 150 m*M* and 1 *M*. Eluted fractions were analysed by SDS–PAGE. The 130 and 150 m*M* fractions were pooled, dialysed into 25 m*M* HEPES pH 8, 300 m*M* NaCl, 10%(*v*/*v*) glycerol and concentrated by ultrafiltration (Amicon, 10 kDa cutoff) to a final concentration of 10 mg ml^−1^.

### 2.2. Crystallization   

Crystals were obtained by sitting-drop vapour diffusion using the commercial JCSG-*plus* screen (Molecular Dimensions) in a 96-well format and using a liquid-handling robot (Mosquito, TTP Labtech) to pipet drops of 150 nl protein solution plus 150 nl reservoir solution. Diffraction-quality crystals appeared over a reservoir consisting of 0.2 *M* ammonium citrate dibasic, 20%(*w*/*v*) polyethylene glycol 3350. In preparation for X-ray diffraction experiments, crystals were immersed for a few seconds in reservoir solution supplemented with 10%(*v*/*v*) ethylene glycol, mounted in nylon loops and quenched in liquid nitrogen.

### 2.3. Structure determination   

X-ray diffraction data were recorded on beamline I04-1 at Diamond Light Source (Table 1 ▸). Initial phases were obtained by molecular replacement with *Phaser* (McCoy *et al.*, 2007 ▸) using diffraction data to 3.5 Å resolution and the structure of the monomer of 3-hydroxybutyryl-CoA dehydrogenase from *Clostridium butyricum* as a search model (PDB entry 4kue; Kim *et al.*, 2014 ▸). Following alignment between the search and target sequences (43% identity), non-identical side chains were trimmed to the β-carbon using *CHAINSAW* from *CCP*4 (Winn *et al.*, 2011 ▸). Analysis of the Matthews volume suggested that the asymmetric unit could contain up to eight FadB2 monomers. However, the successive placement of six copies of the search model into the asymmetric unit generated a complete structure. *Phaser* *Z*-scores after translation search of 8.9 to 32.3 for copies 1 to 6, respectively, provided confidence that a correct solution had been identified. The σ_A_-weighted 2*F*_o_ − *F*_c_ map showed clear density for many nonconserved side chains, allowing the structural model to be unequivocally completed and refined with *Coot* (Emsley *et al.*, 2010 ▸) and *REFMAC*5 (Murshudov *et al.*, 2011 ▸). Temperature factors were modelled using TLS refinement in combination with individual *B* factors, while noncrystallographic restraints were imposed across the six independent copies in the asymmetric unit. Comparison of ‘medium’ versus ‘loose’ or no NCS restraints produced virtually identical *R* factors and root-mean-square deviations between NCS-related molecules, while employing ‘tight’ restraints increased the free *R* factor from 19% to 20.7%. Therefore, ‘medium’ restraints were adopted for the final refinement. The final model has good stereochemistry (Table 1 ▸) and *R* factors of 19.0% for the working set and 20.1% for the test set. X-ray diffraction data-collection and refinement statistics are reported in Table 1 ▸. Coordinates and structure factors have been deposited in the Protein Data Bank (PDB) with accession code 6hrd.

## 3. Results and discussion   

### 3.1. X-ray crystal structure of *Mtb* FadB2   

**Figure 2:**
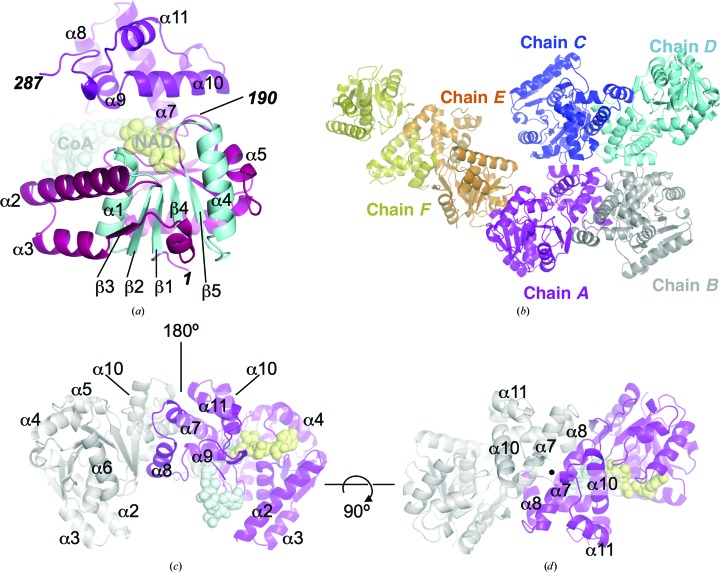
Ribbon representation of the structure of *Mtb* FadB2. (*a*) The FadB2 monomer consists of an N-terminal domain (purple–red) with two Rossmann-fold motifs (β1–α1–β2 and β4–α4–β5; cyan), and an α-helical bundle in the C-terminal domain (α7–α11; magenta). The location of the active site is indicated with translucent spheres for CoA and NAD^+^ (derived from the superposition with ligand-bound HsHAD; PDB entry 1f0y; Barycki *et al.*, 2000 ▸). Secondary-structure elements are labelled according to the secondary-structure analysis by *DSSP* (Kabsch & Sander, 1983 ▸) as shown in Fig. 3 ▸. The locations of the N- and C-termini are indicated with the corresponding residue numbers (1 and 287; bold italics), as is the domain boundary (190). (*b*) Representation of the three FadB2 dimers in the asymmetric unit of the crystal structure. (*c*, *d*) Two orthogonal views of the FadB2 dimer (chains *A* and *B*). The dimer interface is formed entirely by the helical C-terminal domain (helices α7–α11).

In a previous study, we characterized the enzymology of *Mtb* FadB2 (Rv0468) and showed that this enzyme catalyses the oxidation of 3-hydroxyacyl-CoA to acetoacetyl-CoA (Taylor *et al.*, 2010 ▸). We have now determined the crystal structure of apo *Mtb* FadB2 at 2.1 Å resolution (Table 1 ▸; Supplementary Fig. S1). The structural model represents amino-acid residues 3–286 of FadB2 (Fig. 2 ▸ *a*) and does not include the disordered N-terminal hexahistidine purification tag. The asymmetric unit contained six protomers of FadB2 assembled into three dimers (Fig. 2 ▸ *b*). Given the resolution limit and the number of unique copies, noncrystallographic symmetry restraints were applied during coordinate and temperature-factor refinement.

The overall structure of the FadB2 monomer (Fig. 2 ▸ *a*) conforms closely to the paradigm of previously solved monofunctional l-3-hydroxylacyl-CoA dehydrogenases. The enzyme presents a two-domain architecture consisting of an N-terminal domain containing a dual Rossmann fold (residues 3–189) and a smaller C-terminal domain (residues 190–286; Figs. 2 ▸ *a* and 3 ▸). The duplicated βαβ motif of the Rossmann fold consists of the secondary-structure elements β1–α1–β2 and β4–α4–β5 (Fig. 2 ▸ *a*). Helices α2, α3 and strand β3 are spliced in between the two βαβ motifs. Strand β6, strand β7 and (the antiparallel) strand β8 complete the central parallel β-sheet, with the latter marking the C-terminal boundary of the N-terminal domain. The C-terminal domain consists of a five-helix bundle (helices α7–α11; Fig. 2 ▸ *a*). The domain boundary is located in the loop between strand β8 and helix α7 (Figs. 2 ▸ *a* and 3 ▸). The active site is located in the cleft between the N- and C-terminal domains.

FadB2 crystallized as a dimer (Figs. 2 ▸ *c* and 2 ▸ *d*), consistent with the structures of human mitochondrial HAD (PDB entry 1f0y; Barycki *et al.*, 2000 ▸) and of *C. butyricum* HAD (PDB entry 4kue; Kim *et al.*, 2014 ▸). The dimer interface is formed solely by the C-terminal domain. The solvent-accessible surface (SAS) buried upon dimerization of FadB2 is ∼1500 Å per monomer. The interface-analysis software *PISA* (Krissinel, 2015 ▸) calculated a complex-formation significance score (CSS) of 0.94, suggesting that the dimer is stable in the solution state. While we did not experimentally verify the oligomeric state of FadB2 in solution, both analysis of the dimer interface and the self-assembly of the structural homologues suggest that FadB2 functions as a dimer *in vivo*.

### 3.2. Comparison with other FadB structures   

FadB2 is a monofunctional enzyme that mediates 3-hydroxyacyl-CoA dehydrogenase activity (Taylor *et al.*, 2010 ▸) and superimposes closely with the corresponding human enzyme, mitochondrial HAD (PDB entry 1f0y; Barycki *et al.*, 2000 ▸), aligning 188 C^α^ atoms with a root-mean-square deviation (r.m.s.d.) of 1.16 Å. There is also a close match with the structure of *C. butyricum* HAD (PDB entry 4kue; Kim *et al.*, 2014 ▸; 277 C^α^ atoms, r.m.s.d. of 0.99 Å). Both orthologues also crystallized as dimers.

In contrast, the multi-catalytic *Mtb* FadB combines an N-terminal enoyl-CoA hydratase domain with a C-terminal HAD segment (Fig. 4 ▸ *a*). Furthermore, FadB forms a non­covalent, heterotetrameric complex with the dimeric ketoacyl-CoA thiolase FadA (PDB entry 4b3h; Venkatesan & Wierenga, 2013 ▸; Supplementary Fig. S2). Thus, this complex mediates three of the four reaction steps in β-oxidation (Fig. 1 ▸), and there is evidence for substrate channelling between the catalytic sites of the complex (Eaton *et al.*, 1996 ▸; Ishikawa *et al.*, 2004 ▸). Similar multifunctional complexes have been described in rat (Kasaragod *et al.*, 2010 ▸), the model plant *Arabidopsis thaliana* (Arent *et al.*, 2010 ▸) and the prokaryote *Pseudomonas fragi* (Ishikawa *et al.*, 2004 ▸).

**Figure 3:**
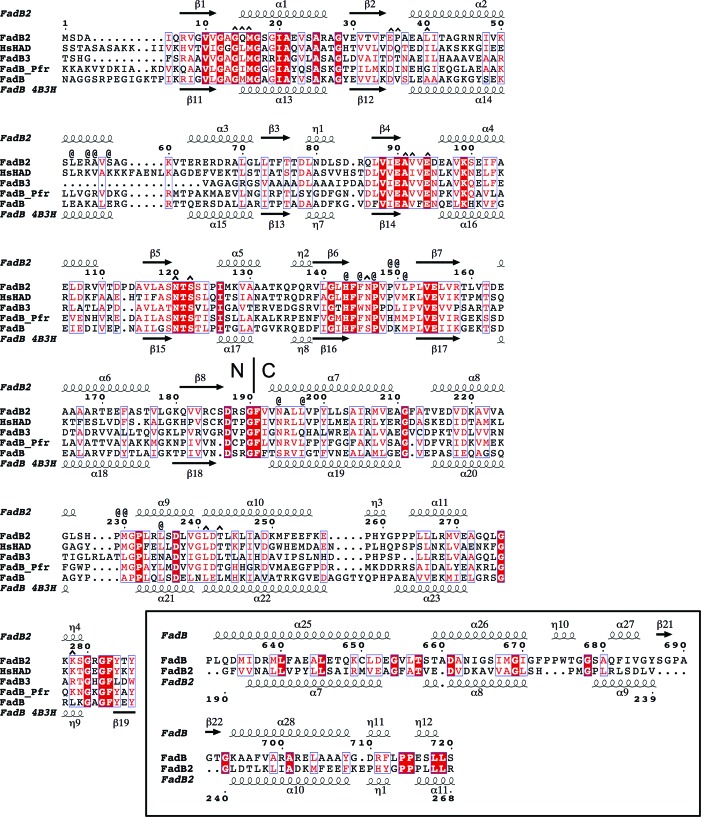
Multiple sequence alignment of FadB homologues. Secondary-structure elements of *M. tuberculosis* FadB2 and FadB (determined by *DSSP*; Kabsch & Sander, 1983 ▸) are indicated above and below the sequences, respectively, with α, β and η indicating α-helices, β-strands and 3_10_-helices, respectively. The boundary between the N- and C-terminal domains is indicated as N | C. Conserved residues are highlighted in red text surrounded by blue boxes; residues that are identical across the alignment are shown in white on a red background. The @ and ^ symbols indicate residues in human l-3-hydroxyacyl-CoA dehydrogenase (HsHAD; PDB entry 1f0y; Barycki *et al.*, 2000 ▸) within a 4.0 Å radius of the CoA and NAD^+^ ligands, respectively. FadB2, *M tuberculosis* FadB2 (Rv0468); FadB3, *M tuberculosis* FadB3 (Rv1715); FadB_Pfr, *Pseudomonas fragi* fatty-acid oxidation complex FadB (α-­subunit; WP_010656816); FadB, *Mtb* trifunctional fatty-acid oxidation protein complex (Rv0860; PDB entry 4b3h; Venkatesan & Wierenga, 2013 ▸). Inset: alignment of C-terminal residues 629–720 of FadB with residues 190–268 of FadB2 as derived from the structural superposition shown in Fig. 4 ▸(*e*).

**Figure 4:**
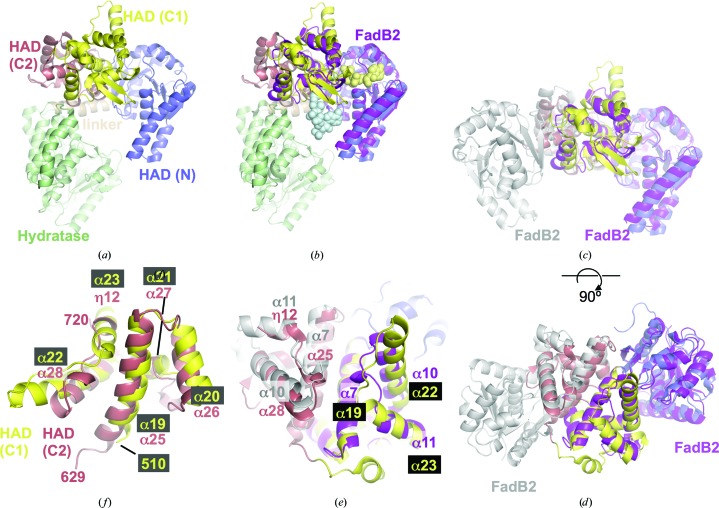
Structural comparison between FadB and the FadB2 dimer. (*a*) Ribbon diagram of *Mtb* FadB (PDB entry 4b3h; Venkatesan & Wierenga, 2013) coloured according to its structural domains. HAD, 3-hydroxyacyl dehydrogenase domain. (*b*) The same as in (*a*), but superimposed on *Mtb* FadB2 (magenta) by secondary-structure matching. Light blue and yellow spheres indicate the location of the active site in the HAD domain. (*c*, *d*) Adding the second protomer of the FadB2 dimer demonstrates the superposition of the FadB2 C-terminal domain (grey) with the HAD-C2 domain of FadB. The FadB hydratase domain is omitted from the view. (*e*) Close-up view of the central area of the FadB2 dimer and its match with the HAD-C1 and HAD-C2 domains of FadB. (*f*) Superposition of the HAD-C1 and HAD-C2 domains of FadB. Residues numbers of the N- and C-terminal residues of the structural fragments are indicated in addition to secondary-structure labels.

When FadB2 is aligned with FadB by secondary-structure matching, FadB2 overlaps with the C-terminal HAD segment of *Mtb* FadB. In this superposition, the N- and C-terminal domains of FadB2 match the HAD-N and HAD-C1 domains of FadB, respectively (Fig. 4 ▸ *b*), while the HAD-C2 domain of FadB remains without a counterpart. However, when the FadB monomer is superimposed onto the FadB2 dimer, the C-terminal domain of the second monomer matches the helices of the HAD-C2 domain of FadB (Figs. 4 ▸ *c*, 4 ▸ *d*, 4 ▸ *e* and the inset in Fig. 3 ▸). Furthermore, the HAD-C1 and HAD-C2 domains are superimposable onto each other (57 C^α^ atoms aligned, r.m.s.d. of 1.55 Å; Fig. 4 ▸ *f*), suggesting that the HAD-C1 and HAD-C2 domains may have arisen from gene duplication.

In the structural context of FadB, the HAD-C2 domain has no obvious role in either catalysis or assembly of the trifunctional FadA–FadB heterotetramer (Supplementary Fig. S2). The largest share (∼75%) of the surface area buried in the interface with FadA is contributed by the hydratase domain of FadB, complemented by the contacts of eight residues in the HAD-C1 domain (∼21% of the surface buried in the interface), while only three residues of the HAD-C2 domain contact FadA. Similarly, the location of the HAD-C2 domain relative to the substrate-binding or cofactor-binding sites does not suggest that the HAD-C2 domain can contact the ligands. Therefore, it is not immediately clear what drove the apparent gene-duplication event that gave rise to the C2 domain. Since the five-helix bundle of the C-terminal domain mediates the dimerization of FadB2, one wonders whether FadB could self-assemble into dimers if the HAD-C2 domain were not present. Indeed, when omitting this domain, it is possible to construct a FadB dimer in analogy to the FadB2 dimer (*i.e.* mediated by dimerization of the HAD-C1 domain), although this hypothetical dimer incurs partial steric overlap between the hydratase domains. Nevertheless, a small rotation about the axis of the linker helix between the hydratase and HAD domains would resolve the overlap. Thus, it appears that an evolutionary driver of integrating the HAD-C2 domain within the polypeptide chain of FadB is to prevent the self-association of FadB into dimers, so that the surface of the hydratase domain remains available for binding to the FadA dimer.

### 3.3. Structural comparison with human mitochondrial HAD and ligand-binding model   

We compared the structure of *Mtb* FadB2 with that of human mitochondrial 3-hydroxyacyl-CoA dehydrogenase (HsHAD), which has been determined in complex with NAD^+^ and acetoacetyl-CoA (PDB entry 1f0y; Barycki *et al.*, 2000 ▸), *i.e.* the product of the 3-hydroxy dehydrogenase reaction. The superposition shows that in the ligand-bound state the N- and C-terminal domains move towards each other in a subtle closing motion around the ligands relative to the apo state of FadB2 (Supplementary Fig. S3*a*).

We identified amino-acid side chains in HsHAD that contact the ligands (4 Å distance cutoff) and we examined the extent to which these contact residues are conserved in FadB2 (Fig. 3 ▸). Of the 15 residues that contact NAD within the distance cutoff in HsHAD, ten are identical in FadB2 and 13 are similar or identical. Similarly, of the 15 residues that contact the CoA ligand in HsHAD, 11 are identical in FadB2 (14 are similar or identical). In percentage terms, this means that the residues predicted to contact the redox cofactor and substrate are 66% or 73% identical, respectively, in FadB2, compared with an overall sequence identity of 40.5% between these two enzymes. Visualization of the structural superposition (Supplementary Fig. S3*b*) illustrates a close match of contacting side chains, despite the subtle difference in overall conformation. For instance, Ser122, which is essential for catalysis (Taylor *et al.*, 2010 ▸), maps onto Ser137 of HsHAD, while the strictly conserved His143 maps onto His158 of HsHAD. Both of these residues are juxtaposed with the nicotinamide group of NAD^+^, indicating the catalytic centre of the enzyme. Overall, the structural analysis of FadB2 in terms of fold, tertiary structure and the conservation of amino acids that are observed to be in contact with the ligands in the human mitochondrial orthologue is entirely consistent with our previous enzymatic characterization (Taylor *et al.*, 2010 ▸).

## 4. Concluding remarks   

The structural features of FadB2 revealed in this study are fully consistent with the enzymatic characterization that we have reported previously (Taylor *et al.*, 2010 ▸). Nevertheless, the extensive redundancy of genes implicated in β-oxidation (and other pathways of fatty-acid metabolism) poses the question of the functional role of FadB2 and how it may work with other enzymes implicated in β-oxidation. The structural comparison of monofunctional FadB2 with the trifunctional FadA–FadB complex provides clues with regard to functional differences. Firstly, the multi-catalytic FadB cannot dimerize in the fashion of the monofunctional enzyme; secondly, FadB2 cannot replace FadB in the trifunctional FadA–FadB complex as it lacks the hydratase domain. This does not preclude the action of FadB2 on products of the hydratase activity of FadB, but given the evidence for substrate channelling within the trifunctional complex, it is unlikely to be the dominant event. Instead, FadB2 may preferentially interact (physically and/or functionally) with other homologues of the *fad* gene family. Interrogation of the STRING database (Szklarczyk *et al.*, 2017 ▸), which computes protein-interaction networks by combining experimental data with database information and homology relationships, suggests the enoyl-CoA hydratase EchA8 and several thiolases of the FadA family as potential interaction partners (Supplementary Fig. S5). These enzymes catalyse the reaction steps preceding and subsequent to 3-hydroxy dehydrogenation, respectively (Fig. 1 ▸).

## 5. Related literature   

The following references are cited in the supporting information for this article: Webb & Sali (2017 ▸) and Zimmermann *et al.* (2018 ▸).

## Supplementary Material

Supplementary Figs. S1-S5.. DOI: 10.1107/S2059798318017242/mn5119sup1.pdf

PDB reference: FadB2, 6hrd

## Figures and Tables

**Table 1:** X-ray diffraction data-collection and refinement statistics Values in parentheses are for the highest resolution shell.

X-ray diffraction data	
PDB entry	6hrd
Beamline	I04-1, Diamond Light Source
Wavelength (Å)	0.92819
Space group	*P*4_3_
*a*, *b*, *c* (Å)	90.22, 90.22, 284.7
Molecules per asymmetric unit	6
Resolution (Å)	90.2–2.11 (2.16–2.11)
*R*_merge_ † (%)	9.9 (104)
Total reflections	881634
Unique reflections	129955
〈*I*/σ(*I*)〉	13.8 (1.6)
Completeness (%)	100 (100)
Multiplicity	6.8 (5.7)
CC_1/2_ †	0.998 (0.583)
Refinement	
Resolution range	90.22–2.11
Unique reflections	123371
*R*_cryst_/*R*_free_ (%)	19.0/22.1
No. of non-H atoms	
Total	13117
Protein	12675
Solvent	424 [water], 18 [glycerol]
R.m.s.d., bonds (Å)	0.013
R.m.s.d., angles (°)	1.63
*B* factors (Å^2^)	
Wilson	39.9
Average overall	42.2
Average protein	42.4
Average solvent	36.4
R.m.s.d. for *B* factors	1.18
Ramachandran plot‡	
Favoured region (%)	96.7
Disallowed (%)	0.18 [3 residues]

† CC_1/2_ (the correlation between half data sets) is defined in Karplus & Diederichs (2012 ▸).

‡ Ramachandran plot statistics were calculated using *MolProbity* (Chen *et al.*, 2010 ▸).
