# Supplementary material for: Crystal structure of Mycobacterium tuberculosis FadB2 implicated in mycobacterial β-oxidation
Source: Acta Crystallogr D Struct Biol. 2019 Jan 8;75(Pt 1):101–8. doi: 10.1107/S2059798318017242 (PMC6333283; doi:10.1107/S2059798318017242)
Supplement: Supplementary file 1 [file d-75-00101-sup1.pdf]

STRUCTURAL
BIOLOGY

Volume 75 (2019)

Supporting information for article:

Crystal structure of *M. tuberculosis* FadB2 implicated in mycobacterial β-oxidation

Jonathan A. G. Cox, Rebecca C. Taylor, Alistair K. Brown, Samuel Attoe, Gurdyal S. Besra and Klaus Fütterer

Figure S1

Electron density of FadB2 (chain D). (a) Cα trace of chain D of FadB2 with σ_A-weighted 2Fo-Fc density map (1.5 σ contour level) in the orientation of Fig. 2*a*. Selected secondary structure elements are labelled. (b) Representative section of the electron density map around the C-terminal domain of FadB2 (chain D), with 2Fo-Fc map (blue, 1.5σ) and Fo-Fc maps (green 3.0σ, red -3.0σ). The maps shown were calculated with phases from the final refined model.

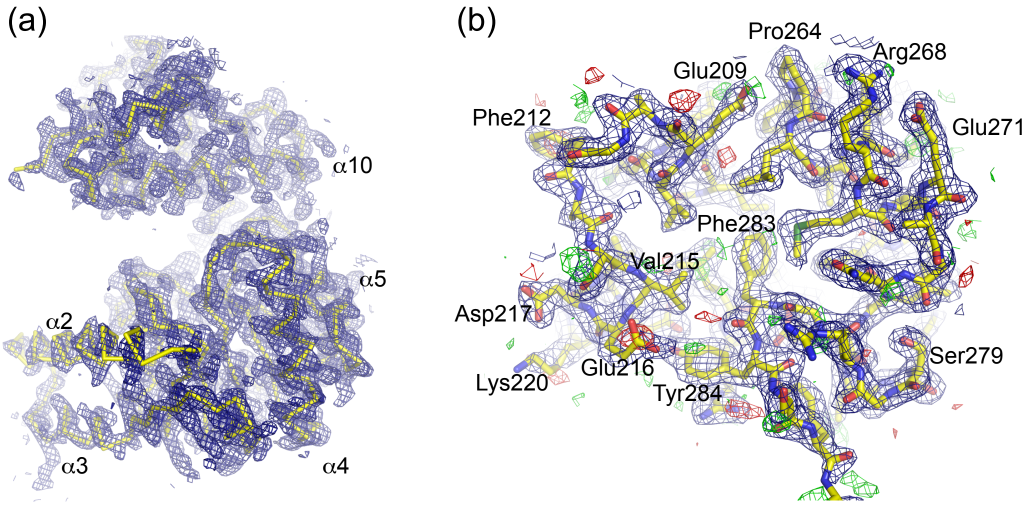

Figure S2

Ribbon representation of the hetero-tetrameric *M. tuberculosis* FadA:FadB complex (PDB entry 4B3H, Venkatesan & Wierenga, 2013), with colouring highlighting the distinct structural domains of FadB. HAD, hydroxyacyl-CoA dehydrogenase; N, N-terminal domain; C1, C2, C-terminal domains 1 and 2.

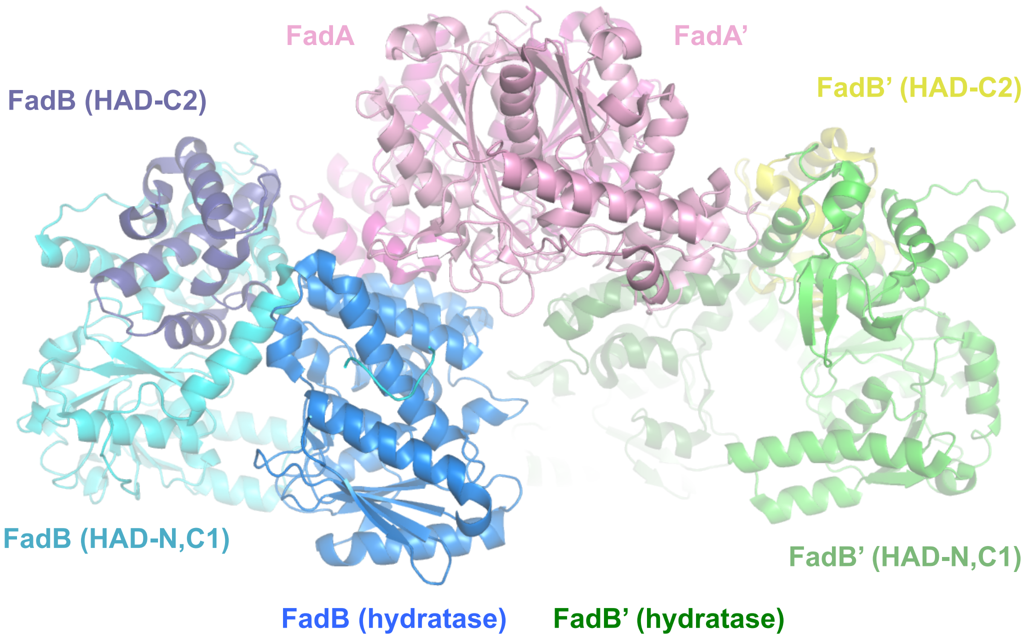

Figure S3
Superposition of ligand-bound human mitochondrial 3-hydroxyacyl CoA dehydrogenase (HsHAD, PDB 1F0Y (Barycki *et al.*, 2000)) with *Mtb* FadB2. (a) Ribbon representations of *Mtb* FadB2 (magenta) and of HsHAD (lime coloured) bound to acetoacetyl-CoA (pale blue spheres) and NAD^+ (pale yellow spheres). b) Close-up view of the catalytic centre of HsHAD, displaying side chains of HsHAD (carbon atoms in lime colour) that contact the ligands (4 Å distance cut-off), and demonstrating the close match of ligand contact residues in *Mtb* FadB2 (carbon atoms in violet). Residues in *Mtb* FadB2 identical to contact residues of HsHAD are indicated with their *Mtb* residue number (cf. Figure 3).

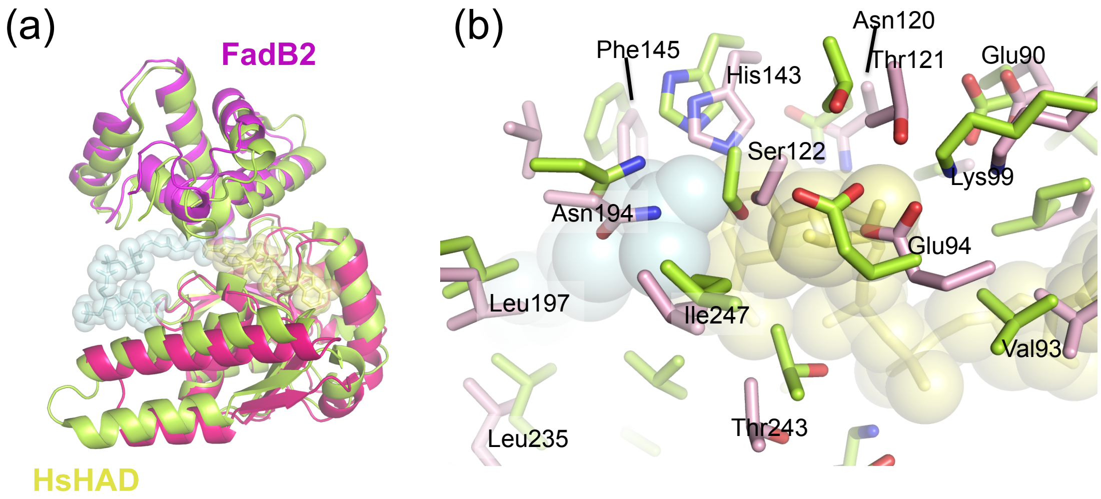

Figure S4

Superposition of the Rossmann-fold motifs of FadB2 with the modelled structure (HHpred,(Zimmermann *et al.*, 2018), Modeller (Webb & Sali, 2017)) of FadB5. The ribbon representing the FadB5 backbone is rainbow-coloured N- (blue) to C-terminus (red), while *Mtb* FadB2 is in grey. The alignment was generated manually in Pymol and optimised using least squares refinement in Swiss PDB Viewer. The secondary structure elements of the Rossmann fold motif are indicated (with respect to *Mtb* FadB2).

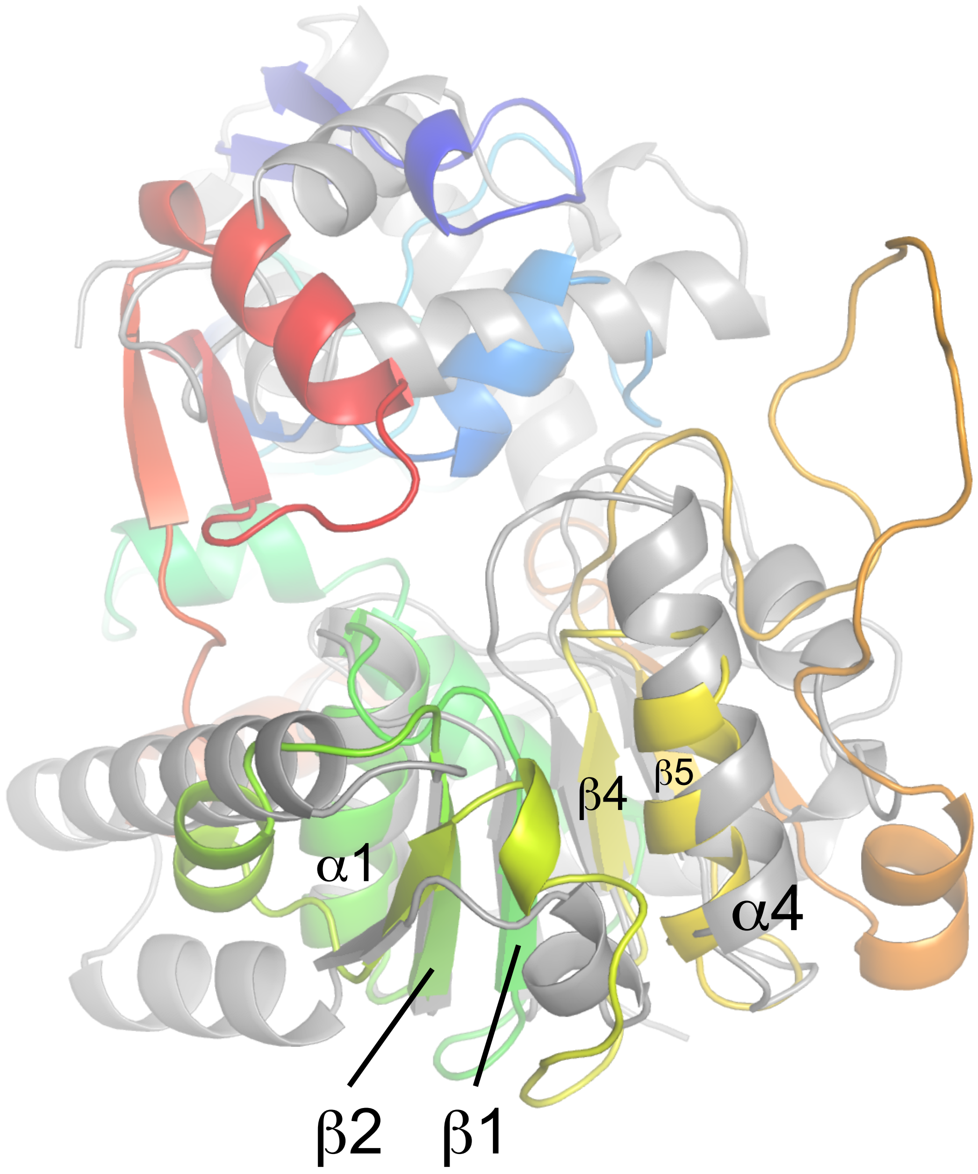

Figure S5

Predicted protein-protein associations of FadB2 as calculated by STRING (Szklarczyk *et al.*, 2017). Sources for connections between nodes in the diagram are colour-coded as per the legend below.

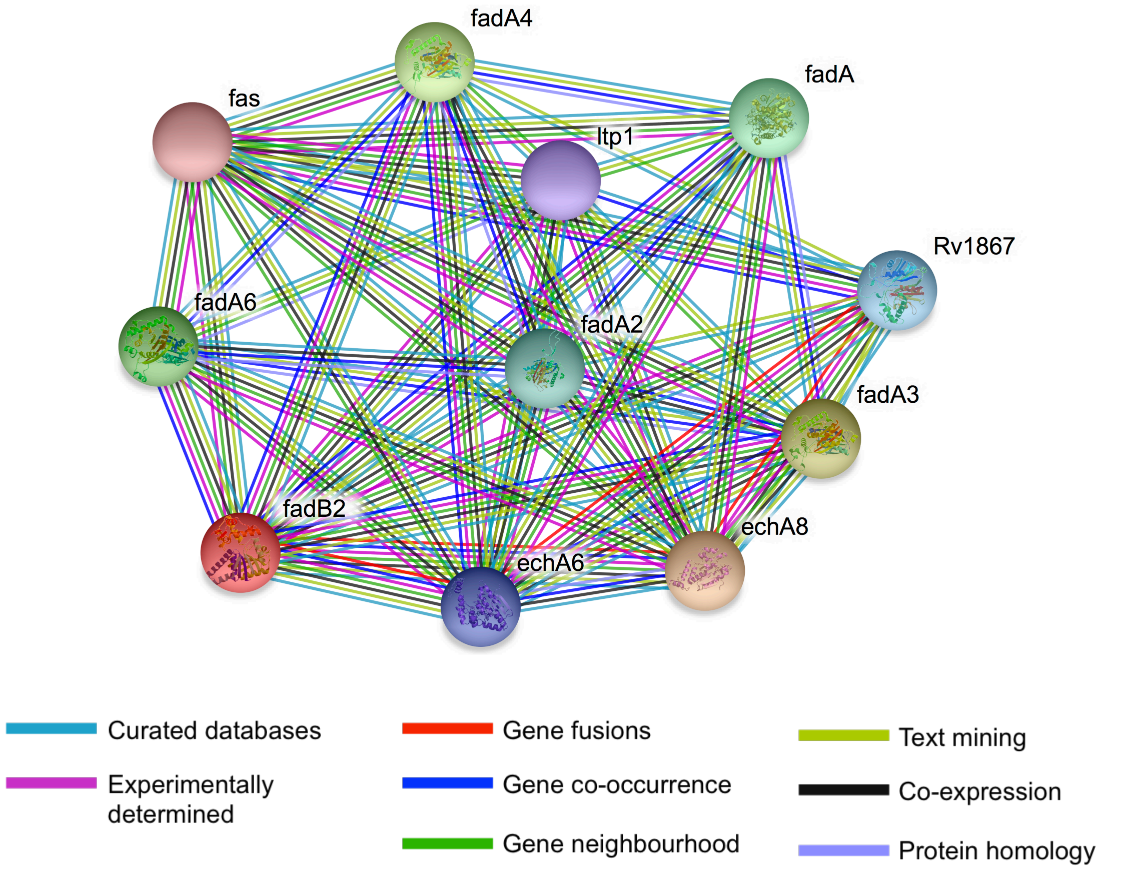

References

Barycki, J. J., O'Brien, L. K., Strauss, A. W. & Banaszak, L. J. (2000). *J Biol Chem* **275**, 27186-27196.

Szklarczyk, D., Morris, J. H., Cook, H., Kuhn, M., Wyder, S., Simonovic, M., Santos, A., Doncheva, N. T., Roth, A., Bork, P., Jensen, L. J. & von Mering, C. (2017). *Nucleic Acids Res* **45**, D362-D368.

Venkatesan, R. & Wierenga, R. K. (2013). *ACS Chem Biol* **8**, 1063-1073.

Webb, B. & Sali, A. (2017). *Methods Mol Biol* **1654**, 39-54.

Zimmermann, L., Stephens, A., Nam, S. Z., Rau, D., Kubler, J., Lozajic, M., Gabler, F., Soding, J., Lupas, A. N. & Alva, V. (2018). *Journal of molecular biology* **430**, 2237-2243.
